# Construction and analysis of macrophage infiltration related circRNA-miRNA-mRNA regulatory networks in hepatocellular carcinoma

**DOI:** 10.7717/peerj.10198

**Published:** 2020-10-20

**Authors:** Yuhan Chen, Yalin Li, Guanglei Zheng, Peitao Zhou

**Affiliations:** 1 Department of Radiation Oncology, Nanfang Hospital, Southern Medical University, Guangzhou, China; 2 The First School of Clinical Medicine, Southern Medical University, Guangzhou, China

**Keywords:** Circular RNA, Regulatory network, Macrophage, Hepatocellular carcinoma

## Abstract

**Background:**

Macrophage play a crucial role in regulating tumor progression. This study intended to investigate the circular RNA (circRNA) regulatory network associated with macrophage infiltration in hepatocellular carcinoma (HCC).

**Methods:**

The immune cell fractions of HCC from The Cancer Genome Atlas (TCGA) and International Cancer Genome Consortium were calculated by Estimation of the Proportion of Immune and Cancer cells algorithm. The differentially expressed mRNAs (DEmRNAs), microRNAs (DEmiRNAs) and circRNAs (DEcircRNAs) were identified from HCC and adjacent non-tumor cases of TCGA or Gene Expression Omnibus database. The DEmRNAs related to macrophage were selected by weighted gene co-expression network analysis and then utilized to generate the circRNA-miRNA-mRNA network. A hub circRNA regulatory network was established based on the co-expressed DEmiRNAs and DEmRNAs owning contrary correlation with the clinical characteristics, survival and macrophage infiltration level. A gene signature based on the DEmRNAs in hub network was also generated for further evaluation. The circRNA binding bite for miRNA was detected by luciferase assay.

**Results:**

High macrophage fraction predicted good survival for HCC. A circRNA-miRNA-mRNA network was constructed by 27 macrophage related DEmRNAs, 21 DEmiRNAs, and 15 DEcircRNAs. Among this network, the expression of hsa-miR-139-5p was negatively correlated with CDCA8, KPNA2, PRC1 or TOP2A. Hsa-miR-139-5p low or targeted DEmRNA high expression was associated with low macrophage infiltration, high grade, advanced stage and poor prognosis of HCC. Additionally, the risk score generated by 4-DEmRNA signature could reflect the macrophage infiltration status and function as an independent prognostic factor for HCC. Finally, hsa_circ_0007456 acting on hsa-miR-139-5p related network was viewed as the hub circRNA regulatory network. Taken together, some circRNA regulatory networks may be associated with macrophage infiltration, which provides clues for mechanism study and therapeutic strategies of HCC.

## Introduction

Hepatocellular carcinoma (HCC) is one of the most common malignant tumors in the world, accounting for 75%–85% of primary liver cancers (Bray et al., 2018). Despite the great advance of the diagnosis and therapeutic strategies for HCC, the prognosis still needs to be further improved. Therefore, there is an urgent need to develop sensitive and specific biomarkers and therapeutic targets for the early diagnosis and treatment of HCC (Yang et al., 2019).

Hepatic macrophages play an important role in maintaining liver homeostasis and the occurrence and development of liver diseases (Heymann & Tacke, 2016). According to the source, macrophages in the liver can be divided into self-renewing tissue-resident phagocytes, namely Kupffer cells, and recruited myeloid monocyte-derived macrophages (Dou et al., 2019). Hepatic macrophages are essential in the pathogenesis of HCC. They can not only establish a pro-inflammatory microenvironment for tumorigenesis, but also play an anti-tumor immune surveillance function under specific conditions (Tian et al., 2019). There is increasing evidence support that hepatic macrophages may become a prognostic factor in patients with HCC (Li et al., 2009).

Circular RNA (circular RNA, circRNA) is a class of covalently closed single-stranded circular RNA molecules formed by back-splicing (Jeck et al., 2013). Many studies have confirmed that circRNA is closely related to the occurrence and development of tumors, suggesting that circRNA has application value as a diagnostic marker and prognostic factor. CircRNA serve as a microRNA (miRNA) sponge to participate in the regulation of gene expression during the progression of various cancers (Lei et al., 2018). However, the circRNA-miRNA-mRNA regulatory network related to macrophage infiltration in HCC is still unclear.

The design of this research is shown in Fig. S1. We calculated the fraction of infiltrating immune cells, cancer-associated fibroblasts (CAFs) and endothelial cells in HCC patients through the Estimation of the Proportion of Immune and Cancer cells (EPIC) algorithm (Racle et al., 2017). We found that the macrophage fraction was related to the survival rate of HCC from The Cancer Genome Atlas (TCGA) and International Cancer Genome Consortium (ICGC) projects. Next, the hub macrophage-related differentially expressed (DE) mRNAs between paired normal and tumor tissues were identified through weighted gene co-expression network analysis (WGCNA) and protein protein interaction (PPI) network analysis. The circRNA-miRNA-mRNA regulatory network was constructed based on these hub macrophage-related DEmRNAs with prognostic value. After co-expression and survival analysis, the co-expressed DEmiRNAs and DEmRNAs owning contrary correlation with the clinical characteristics and survival were selected as the hub subnetwork. In addition, a gene signature based on the DEmRNAs in hub subnetwork was generated for further evaluation. The macrophage fraction was also assessed in HCC with different level of these hub genes or risk score. Finally, the interaction between circRNA and miRNA was detected by luciferase assay and a hub circRNA regulatory network was generated according to the hub miRNA subnetwork. These results suggest some circRNA regulatory networks may play roles in modulating macrophage infiltration in HCC.

## Materials and Methods

### Data collection

In this study, 383 cases (including 50 pairs of HCC and non-tumor cases) and 403 cases (including 199 pairs of HCC and non-tumor cases) of transcriptome data and corresponding clinical information were downloaded from the TCGA data portal (https://tcga-data.nci.nih.gov/tcga/) and the ICGC data portal (https://dcc.icgc.org/), respectively. 392 cases (including 49 pairs of HCC and non-tumor cases) and 166 cases of HCC miRNA expression data and corresponding clinical data were obtained from TCGA and GSE31384 in Gene Expression Omnibus (GEO) database, respectively. The individual survival time of these HCC cases is more than 30 days. In addition, a total of 15 pairs of HCC and non-tumor tissues with circRNA microarray data were downloaded from GSE94508 (five pairs of tissues), GSE97332 (seven pairs of tissues) and GSE78520 (three pairs of tissues). In order to screen out the commonly altered RNAs, we did not distinguish the cause of HCC.

### Cell fractions and survival analysis

According to the method evaluation, EPIC, but not CIBERSORT nor xCell, could generate an absolute score representing the cell fraction(Sturm et al., 2019). Therefore, we used EPIC for analysis. The EPIC algorithm (http://epic.gfellerlab.org/) was utilized to calculate the seven cell fractions, including five immune cells (B cells, CD4 T cells, CD8 T cells, NK cells and Macrophages), CAFs and Endothelial cells in HCC. The association between cell fractions and OS was performed by univariate survival analysis.

### Construction of co-expression networks

The R package “Bioconductor Limma” was used to identify the DEmRNAs from 50 or 199 pairs of tumor and non-tumor cases of TCGA and ICGC projects, respectively. After calculating false discovery rate (FDR) for each gene by Benjamini–Hochberg method, those DEmRNAs with FDR<0.05 and |log2FC|>1 were selected. To identify the DEmRNAs related to macrophage fraction, the R package “Weighted Gene Co-expression Network Analysis (WGCNA)” was performed to construct co-expression networks in both TCGA and ICGC projects. A soft threshold power parameter was calculated by the pickSoft-Threshold function to generate a scale-free network and the data matrix was transformed into topological overlap matrix (TOM). A tree diagram was constructed by hierarchical clustering based on the TOM-based dissimilarity measure. We set soft-thresholding power as 4, scale free R2 as >0.90 and minimal module size as 50 to figure out key modules. The correlation between module eigengenes and clinical trait was calculated by Pearson’s correlation analysis and adjusted *P* < 0.05 was considered significant. Among the significant modules, the module with a significant correlation between the gene significance for macrophage and module membership was identified as the clinically significant module. Those DEmRNAs from TCGA and ICGC projects both in the same clinically significant module were used for the following study.

### Protein protein interaction (PPI) network and hub subnetwork analysis

The PPI network of DEmRNAs were predicted by the Search Tool for the Retrieval of Interacting Genes/Proteins (STRING) database (https://string-db.org/). The hub subnetworks and hub DEmRNAs were obtained through the MCODE plugin of Cytoscape.

### Identification of DEmiRNAs and DEcircRNAs

The method for DEmiRNAs and DEcircRNAs identification was described in our previous study (Zhou et al., 2020). Briefly, the DEmiRNAs were screened out from 49 paired HCC and non-tumor cases from TCGA by using R package “Bioconductor Limma” and the selection criteria was FDR <0.05 and |log2FC |>1. The DEcircRNAs between HCC and non-tumor samples from three GEO datasets were identified by a robust rank aggregation method. Volcano plot and heatmap were used to visualize the DEmiRNA and DEcircRNAs, respectively.

### Generation of macrophage infiltration related circRNA-miRNA-mRNA regulatory network

We used the microRNA Data Integration Portal (MiRDIP) to predict DEmiRNAs targeting mRNAs. The miRNA targets are predicted by integrating the results from more than 20 miRNA-related databases in MiRDIP (Tokar et al., 2018). And we selected those DEmRNAs potentially targeted by DEmiRNAs with the very high score (top 1%). Next, the cancer-specific circRNA database (CSCD, http://gb.whu.edu.cn/CSCD/) was used to predict DEcircRNAs that can bind to DEmiRNAs. Generally, circRNAs do not affect the expression of miRNAs after adsorbing miRNAs. In addition, some miRNAs can inhibit highly expressed mRNAs in an antagonistic up-regulated manner (Chen et al., 2020). Therefore, the choice of circRNA-miRNA or miRNA-mRNA pairs were not restricted by their expression pattern. Then the DEcircRNA-DEmiRNA pairs and the DEmiRNA-DEmRNA pairs were intersected to form the final circRNA-miRNA-mRNA regulatory network. Cytoscape 3.4.0 (http://cytoscape.org/) was used to visualize the regulatory network.

### Construction of the DEmRNA signature for HCC

The correlation between DEmiRNAs and DEmRNAs were evaluated by the Pearson correlation analysis. According to the co-expressed DEmiRNAs and DEmRNAs owning contrary correlation with the clinical characteristics, survival and macrophage infiltration level, the hub DEmiRNAs-DEmRNAs subnetwork was identified. Based on the hub DEmRNAs in the hub subnetwork, we used multivariate Cox regression analysis to construct a 4-DEmRNA signature for predicting the prognosis of HCC patients in both TCGA and ICGC projects. Based on the risk score formula, the expression level of each DEmRNA was multiplied by the corresponding coefficient and then added together to calculate the risk score of each patient.

### Luciferase reporter assay

The wild type or mutated hsa_circ_0007456 sequence containing hsa-miR-139-5p binding site were synthesized and inserted into pmiR-RB-REPORT™ vector (RIBOBIO, Guangzhou, China), respectively. The above vectors and hsa-miR-139-5p mimics or negative control were co-transfected into HEK-293T cells (Shanghai Advanced Research Institute, Chinese Academy of Sciences) using Lipofectamine3000 (Invitrogen). 48 h after transfection, the cells were harvested for firefly and renilla luciferase activities detection by using the dual-luciferase reporter assay system (Promega, Massachusetts,USA). Renilla luciferase served as the internal control for luciferase activity.

### Statistical analysis

OS differences between different groups were evaluated by Cox regression or Kaplan–Meier survival analysis. The differences of gene expression or risk score between each clinicopathological characteristics were assessed by Mann–Whitney-Wilcoxon Test. The differences in macrophage fractions with different gene expression status or risk scores were analyzed by Mann–Whitney-Wilcoxon Test. R software version 3.4.2 was used for statistics, and *P* < 0.05 was considered to have statistical difference.

## Results

### High macrophage fraction correlates with good prognosis of HCC

**Figure 1:**
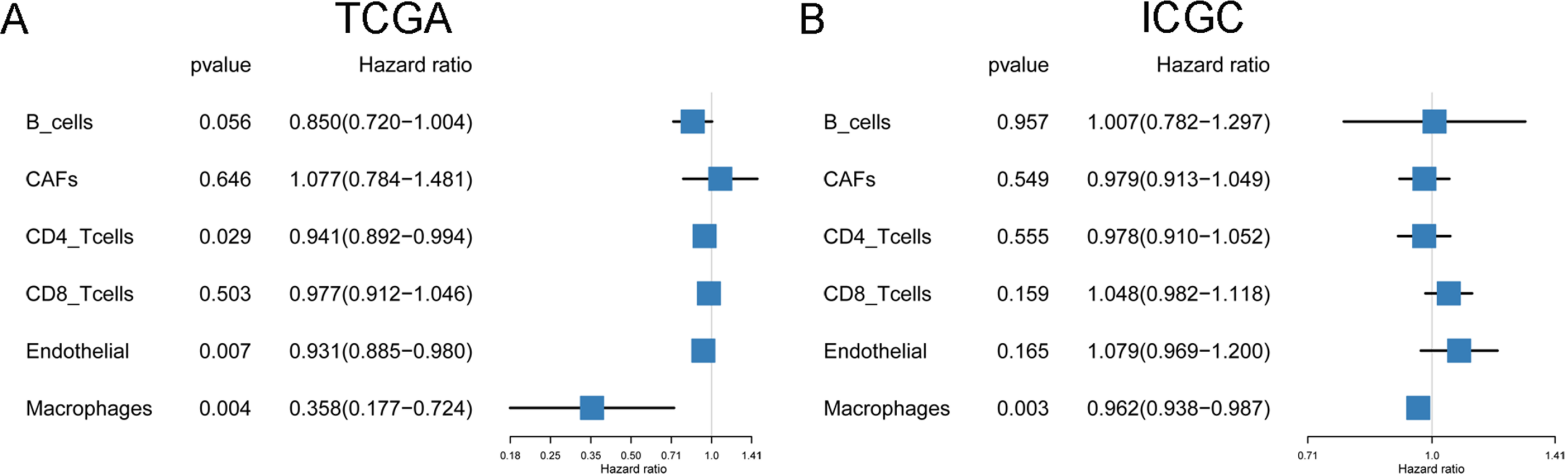
The prognostic value of six cell fractions in HCC patients from TCGA (A) and ICGC (B).

The infiltrations of 7 cell types in TCGA and ICGC HCC patients were evaluated by EPIC. The fraction of NK cells was zero in most patients, thus we excluded NK cells in our analysis. Next, we performed the univariate Cox regression analysis to evaluate the prognostic value of 6 cell fractions for HCC. The results showed that in TCGA project, patients with a high proportion of CD4 T cells, endothelial cells, and macrophages had better OS than those with a low proportion of corresponding cells (Fig. 1A). In ICGC project, only the high macrophage fractions had a protective effect on survival (Fig. 1B). It is suggested that the higher the macrophage fractions, the better the survival of HCC patients, and the infiltration of macrophages may modulate the HCC progression.

**Figure 2:**
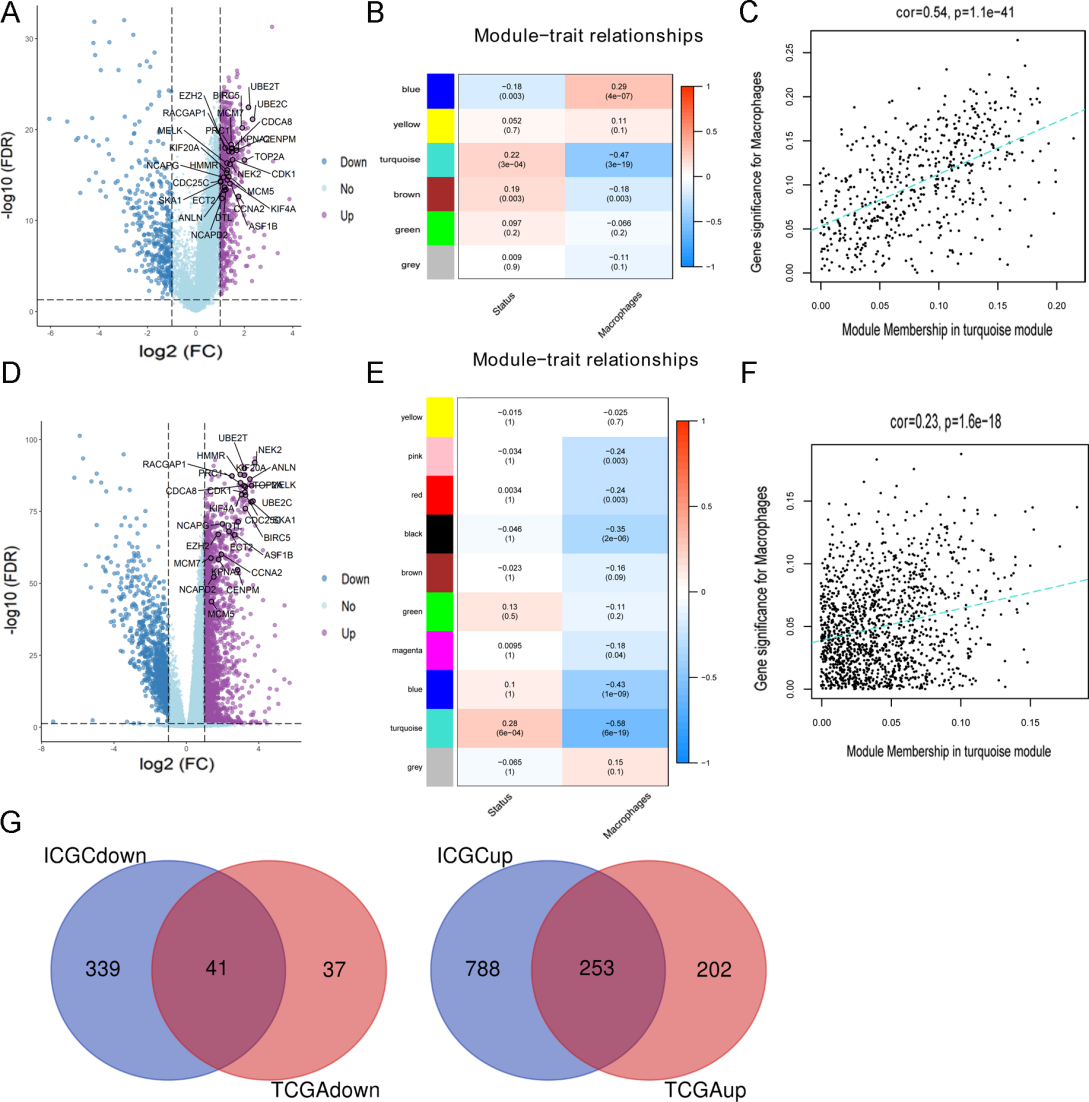
Identification of DEmRNAs related macrophage in HCC. (A) The DEmRNAs were screened out from TCGA. (B) The relationship between module and clinical trait in TCGA by WGCNA. (C) The correlation between the gene significance for macrophage and module membership in TCGA by WGCNA. (D) The DEmRNAs were screened out from ICGC. (E) The relationship between module and clinical trait in ICGC by WGCNA. (F) The correlation between the gene significance for macrophage and module membership in ICGC by WGCNA. (G) Identification of DEmRNAs related macrophage both in TCGA and ICGC.

### Identification of DEmRNAs associated with macrophages

There were 1,180 DEmRNAs (794 up and 386 down) between 50 paired cancer and adjacent tissues of TCGA HCC patients (Fig. 2A). The WGCNA results showed that the turquoise module was significantly negatively correlated with macrophage faction while positively correlated with survival status (Fig. 2B). And there existed a significantly positive correlation between the gene significance for macrophage and module membership (Fig. 2C). In addition, 2809 DEmRNAs (1886 up and 923 down) were identified from 199 paired cancer and adjacent tissues of ICGC HCC cases (Fig. 2D). Similar to the results of TCGA, the turquoise module showed a negative correlation with macrophage faction and positive correlation with survival status (Fig. 2E). And the gene significance for macrophage was significantly correlated with module membership in turquoise module for ICGC project (Fig. 2F). Thus, the turquoise module was considered as the clinically significant module and the DEmRNAs in the turquoise module were extracted for further analysis. Finally, 294 DEmRNAs (253 up and 41 down) associated with macrophage were obtained after taking intersection of the results from TCGA and ICGC (Fig. 2G).

### Construction of macrophage related circRNA-miRNA- mRNA regulatory network in HCC

**Figure 3:**
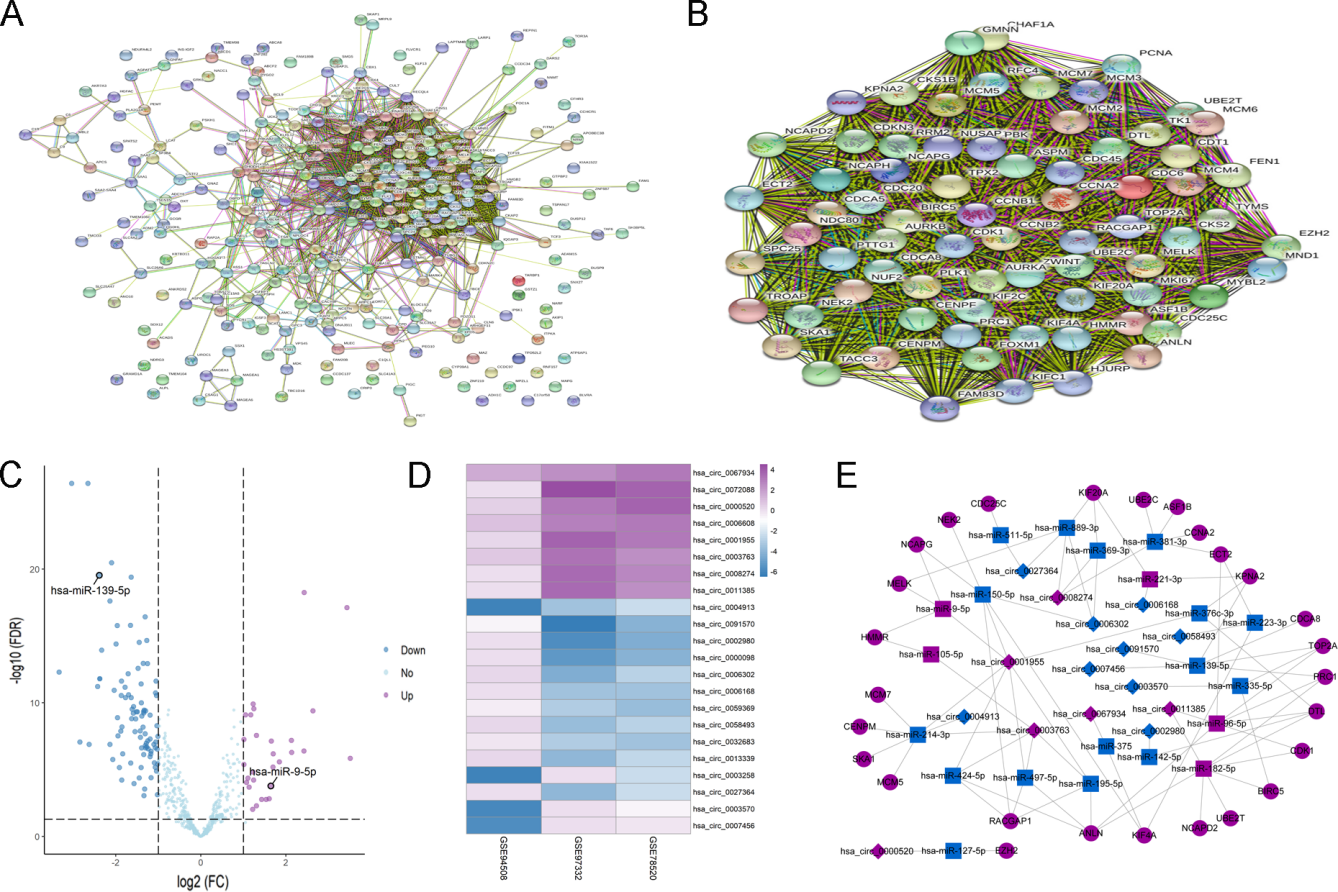
Construction of circRNA-miRNA-mRNA network related to macrophage in HCC. (A) PPI network analysis of 294 DEmRNAs related to macrophage. (B) Identification of 27 DEmRNAs in hub subnetworks of PPI network by MCODE plugin of cytoscape. (C) Identification of DEmiRNAs from TCGA. (D) Identification of DEcircRNAs from 3 GEO datasets. (E) Construction of circRNA-miRNA-mRNA network based on 27 DEmRNAs related to macrophage. The diamond, rectangle and ellipse indicated circRNA, miRNA and mRNA, respectively. Purple and blue represented up- and down-regulated, respectively.

**Figure 4:**
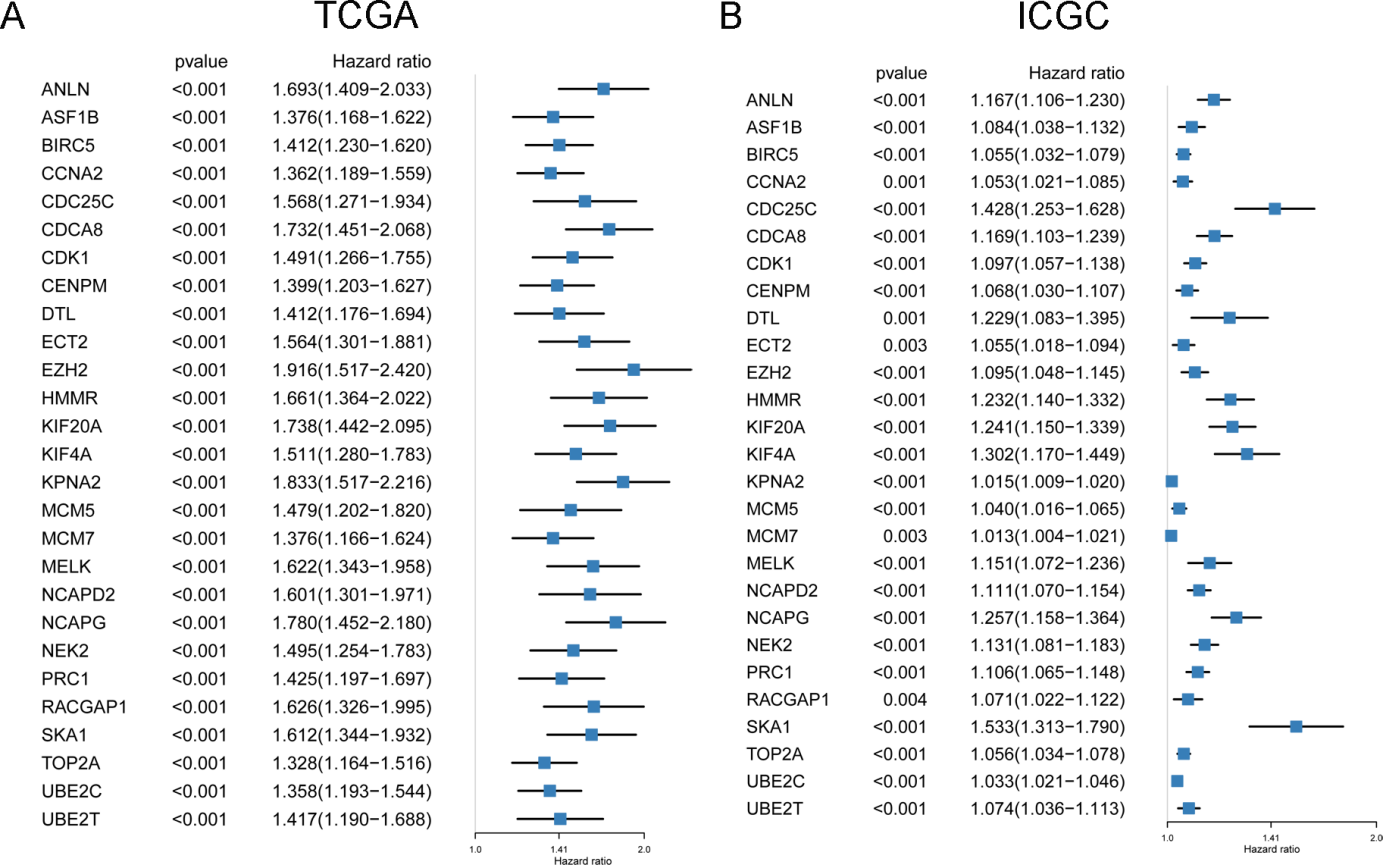
The prognostic value of 27 DEmRNAs related to macrophage in HCC patients from TCGA (A) and ICGC (B).

The potential relationship of 294 DEmRNAs were analyzed through STRING database (Fig. 3A), and a sub-network containing 73 DEmRNAs was obtained through further analysis of the MCODE plugin of Cytoscape (Fig. 3B). Then 121 DEmiRNAs (29 up-regulated, 92 down-regulated) and 22 DEcircRNAs (8 up-regulated, 14 down-regulated) were identified from the paired HCC and non-tumor cases of TCGA and GEO datasets, respectively (Figs. 3C and 3D). By using CSCD and miRDIP database, 1261 pairs of circRNA-miRNA and 31270 pairs of miRNA-mRNA were obtained. After intersecting these RNA pairs, a total of 27 DEmRNAs, 21 DEmiRNAs and 15 DEcircRNAs were used to construct a circRNA-miRNA-mRNA network. The network included 31 pairs of circRNA-miRNA and 54 pairs of miRNA-mRNA (Fig. 3E). In this network, all DEmRNAs were up-regulated in HCC tissues (Figs. 2A and 2D), and associated with the poor prognosis of HCC patients in TCGA (Fig. 4A) and ICGC (Fig. 4B) projects.

### Co-expression and clinicopathological characteristics correlation analysis of DEmiRNAs and DEmRNAs

**Figure 5:**
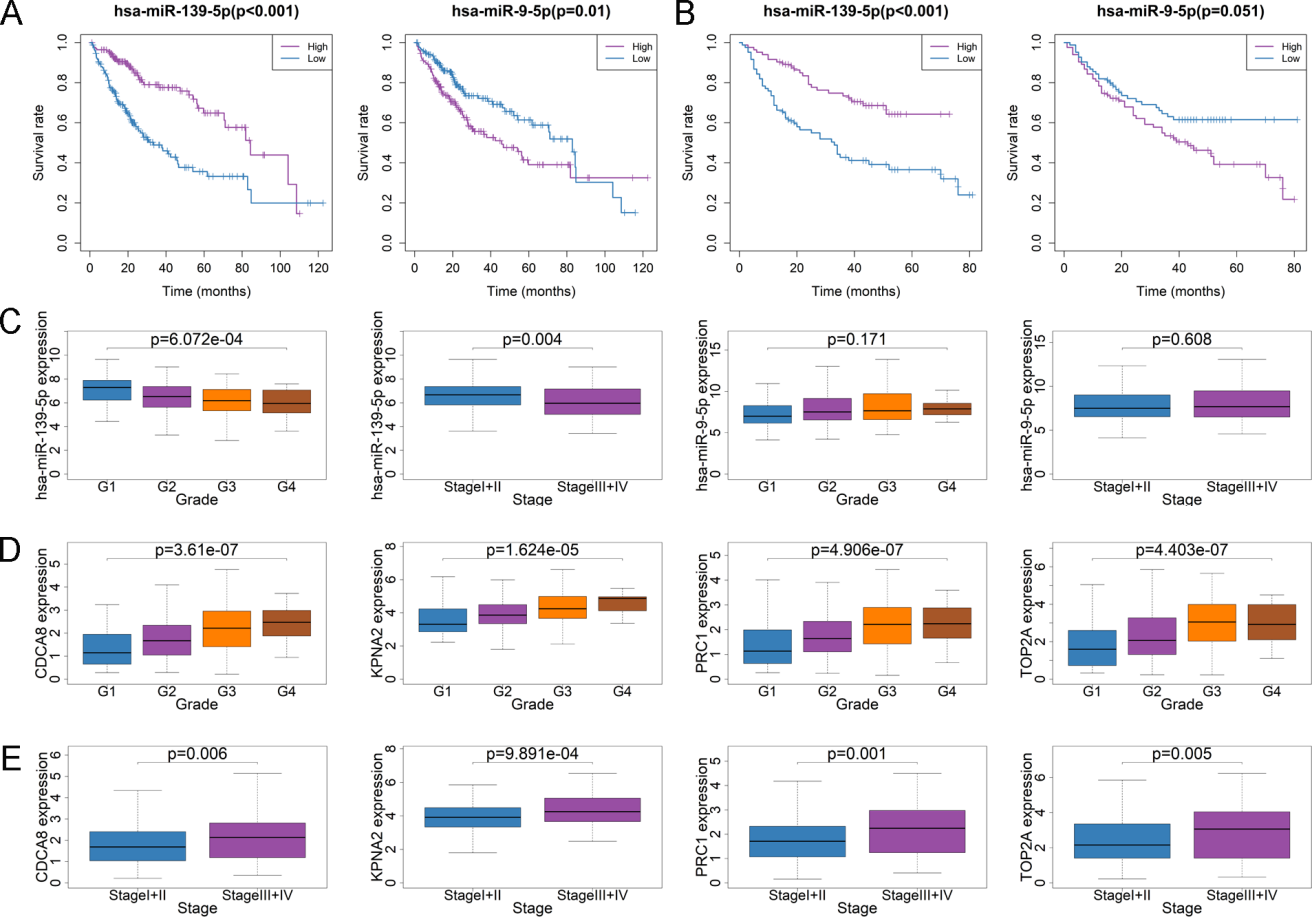
Relationship between DEmiRNA, DEmRNA expression level and clinicopathological characteristics in TCGA. (A) The Kaplan–Meier survival analysis of HCC cases with different hsa-miR-139-5p or hsa-miR-9-5p expression level in TCGA. (B) The Kaplan–Meier survival analysis of HCC cases with different hsa-miR-139-5p or hsa-miR-9-5p expression level in GSE31384. (C) Comparison of hsa-miR-139-5p or hsa-miR-9-5p expression level between different pathological grades and stage. (D) Comparison of CDCA8, KPNA2, PRC1 or TOP2A expression level between different pathological grades. (E) Comparison of CDCA8, KPNA2, PRC1 or TOP2A expression level between different stages.

In order to identify the most potentially interactive miRNA-mRNA pairs, co-expression status between 21 DEmiRNAs and 27 DEmRNAs were performed by Pearson correlation analysis. As shown in Table 1, hsa-miR-139-5p expression was negatively correlated with targeted DEmRNAs, while other DEmiRNAs was positively correlated with DEmRNAs. Since high expression of all DEmRNAs were correlated with the poor survival of HCC patients and high expression of DEmiRNAs are supposed to suppress the effects of targeted DEmRNAs, which indicate that high level of DEmiRNAs must be associated with the good survival of HCC. Among those DEmiRNAs co-expressed with targeted DEmRNAs, the expression level of hsa-miR-139-5p and hsa-miR-9-5p were found to be correlated with survival outcome of HCC. However, only the patients with hsa-miR-139-5p high expression had a longer survival time than those with contrast expression level (Fig. 5A, Fig. S2). Similar results were observed in GSE31384 (Fig. 5B). Additionally, high expression of hsa-miR-139-5p but not hsa-miR-9-5p was significantly correlated with the low pathological grade and TNM stage in TCGA project (Fig. 5C). In line with the negative correlation between the expression of hsa-miR-139-5p and targeted DEmRNAs, the expression of cell division cycle associated 8 (CDCA8), karyopherin alpha 2 (KPNA2), polycomb repressive complex 1 (PRC1) or topoisomerase II alpha (TOP2A) was significantly high in advanced pathological grade of HCC in TCGA project (Fig. 5D). Similarly, the lower the CDCA8, KPNA2, PRC1 or TOP2A expression, the earlier the TNM stage (Fig. 5E). Due to the lack of data about pathological grade in ICGC project, the relationships between CDCA8, KPNA2, PRC1 or TOP2A expression and clinicopathological characteristics could not be fully explored. Except PRC1 and TOP2A, the expression of CDCA8 or KPNA2 was significantly high in the advanced TNM stage of ICGC HCC cases (Fig. S3A).

**Table 1:** The correlation between DEmiRNAs and DEmRNAs evaluated by Pearson correlation analysis.

miRNA	mRNA	R	*P* value
hsa-miR-105-5p	HMMR	0.426092	2.69E−16
hsa-miR-139-5p	CDCA8	−0.48736	1.66E−21
hsa-miR-139-5p	KPNA2	−0.48758	1.58E−21
hsa-miR-139-5p	PRC1	−0.4154	1.72E−15
hsa-miR-139-5p	TOP2A	−0.45648	9.49E−19
hsa-miR-182-5p	ANLN	0.226301	2.75E−05
hsa-miR-182-5p	BIRC5	0.160991	0.003039
hsa-miR-182-5p	CDK1	0.190534	0.000436
hsa-miR-182-5p	DTL	0.158938	0.003439
hsa-miR-182-5p	NCAPD2	0.215005	6.92E−05
hsa-miR-182-5p	PRC1	0.211782	8.93E−05
hsa-miR-182-5p	UBE2T	0.13917	0.010534
hsa-miR-221-3p	KIF20A	0.225893	2.84E−05
hsa-miR-221-3p	KPNA2	0.175684	0.001202
hsa-miR-223-3p	ECT2	0.223804	3.38E−05
hsa-miR-223-3p	KIF4A	0.135483	0.012796
hsa-miR-369-3p	KIF20A	0.185131	0.000637
hsa-miR-376c-3p	KPNA2	0.168853	0.001867
hsa-miR-381-3p	ASF1B	0.139171	0.010533
hsa-miR-381-3p	CCNA2	0.121132	0.026174
hsa-miR-381-3p	ECT2	0.170525	0.001679
hsa-miR-381-3p	UBE2C	0.238382	9.70E−06
hsa-miR-889-3p	KIF20A	0.215462	6.67E−05
hsa-miR-889-3p	MELK	0.123293	0.023599
hsa-miR-9-5p	HMMR	0.303315	1.33E−08
hsa-miR-9-5p	MELK	0.273136	3.53E−07
hsa-miR-9-5p	NCAPG	0.284926	1.03E−07
hsa-miR-96-5p	ANLN	0.21056	9.83E−05
hsa-miR-96-5p	CDK1	0.196134	0.000292
hsa-miR-96-5p	DTL	0.146342	0.007124
hsa-miR-96-5p	ECT2	0.25271	2.62E−06
hsa-miR-96-5p	PRC1	0.201211	0.000201
hsa-miR-96-5p	TOP2A	0.164952	0.002384

### Clinical significance of 4-DEmRNA signature

**Figure 6:**
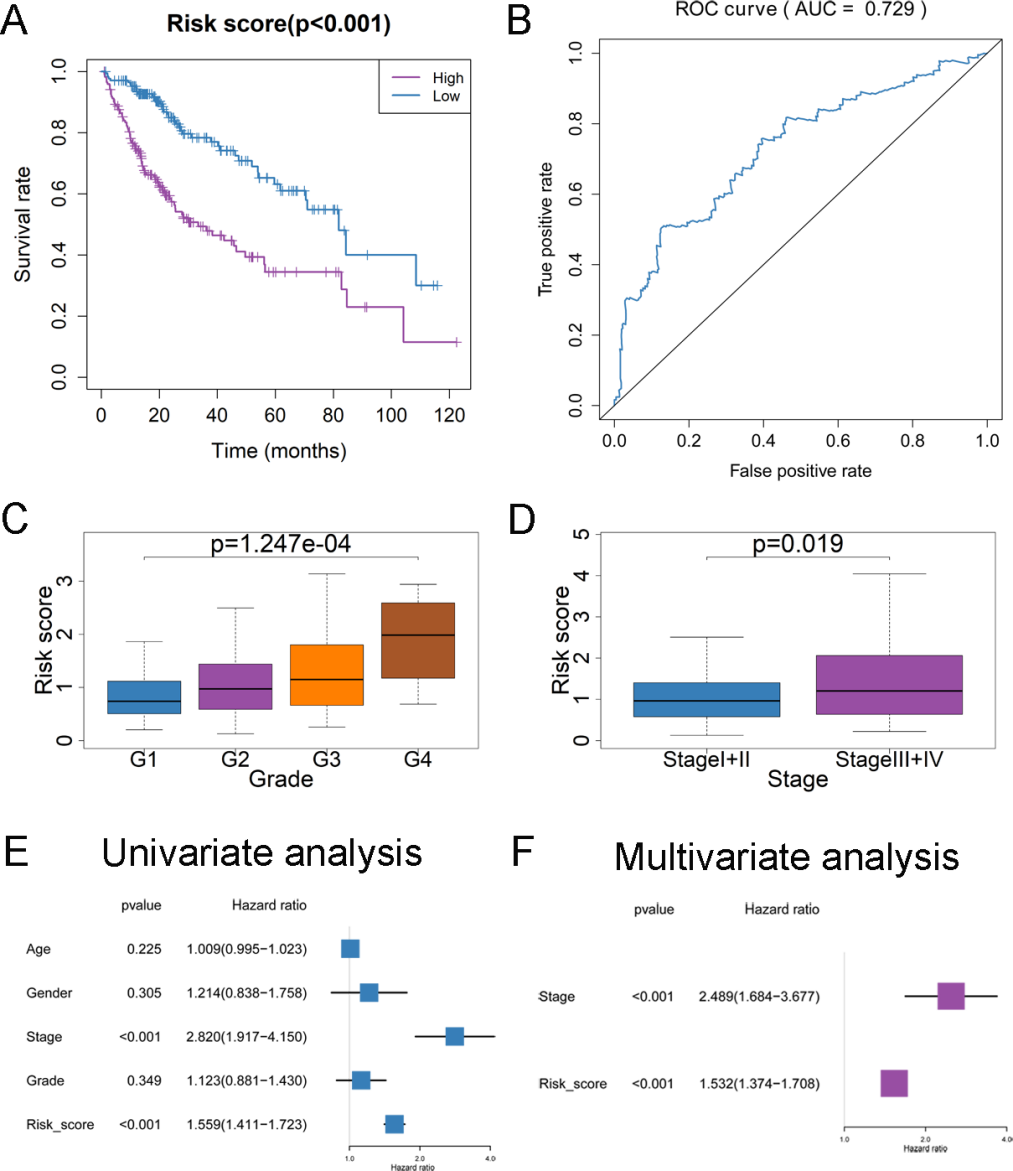
Construction of 4-DEmRNA signature for predicting OS of HCC cases in TCGA. (A) Kaplan–Meier plot of OS of the signature for HCC. (B) The ROC curve of the signature for predicting 3-year survival rate of HCC. (C–D) Comparison of risk score between different pathological grades (C) and stages (D). (E–F) The univariate (E) and multivariate (F) Cox regression analysis of risk score and other clinicopathological characteristics for HCC.

Based on the results that the expression status of CDCA8, KPNA2, PRC1 and TOP2A were correlated with the OS for HCC patients (Fig. S4), we further performed the multivariate Cox regression analysis to construct a 4-DEmRNA signature for predicting the prognosis of HCC patients in TCGA project and validated the prognostic value of 4-DEmRNA signature in ICGC project. The risk score for each patient was calculated according to expression levels of 4 DEmRNAs. Risk score = (2.3198 * CDCA8) + (2.3200 * KPNA2) - (1.5253 * PRC1) - (0.7859 * TOP2A). By using the median risk score as the cutoff value, patients were divided into high- and low-risk group. Patients in the high-risk group had significantly shorter OS than those in the low-risk group both in TCGA and ICGC projects (Fig. 6A, Fig. S3B). The area under curve (AUC) of ROC curve for CDCA8, KPNA2, PRC1, TOP2A or 4-DEmRNA signature from TCGA dataset was 0.688, 0.699, 0.644, 0.646, 0.729, respectively (Fig. S4A, Fig. 6B), which indicated that the signature has a better predictive value than the individual RNAs. The AUC of ROC curve for CDCA8, KPNA2, PRC1, TOP2A or 4-DEmRNA signature from ICGC dataset was 0.781, 0.76, 0.76, 0.741, 0.742, respectively (Fig. S4B, Fig. S3C), which suggested that both signature and individual RNAs have moderate predictive power. Additionally, the risk score was higher in advanced pathological grade or stage both in TCGA (Figs. 6C and 6D) and ICGC (Fig. S3D) projects. Moreover, risk score and stage could serve as the independent prognostic factors for OS both in TCGA (Figs. 6E and 6F) and ICGC (Fig. S3E) projects by multivariate Cox regression analysis.

### Hub circRNA network construction based on hsa-miR-139-5p and 4-DEmRNA signature

**Figure 7:**
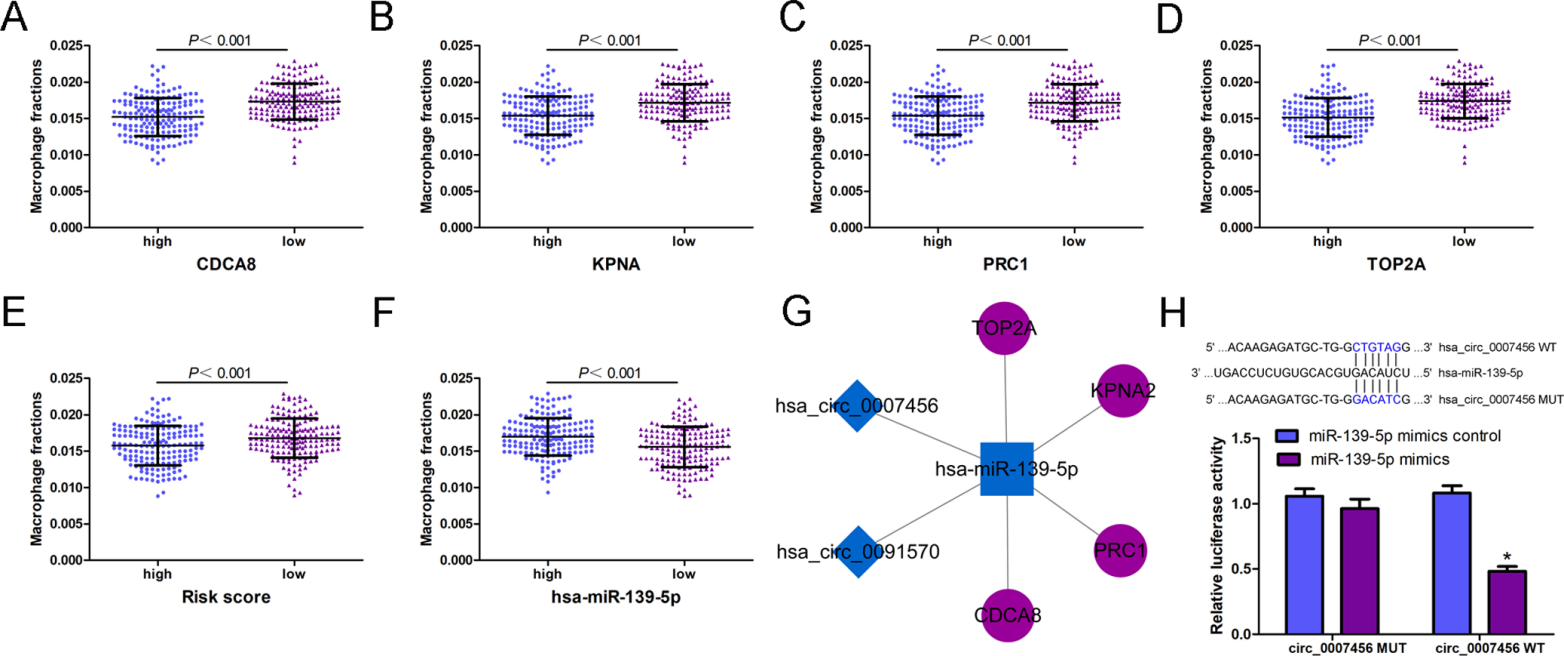
The macrophage fractions in HCC cases with different gene expression level from TCGA. The macrophage fractions in HCC cases with different expression levels of CDCA8(A), KPNA2(B), PRC1(C), TOP2A (D), risk score (E), hsa-miR-139-5p (F). (G) Construction of hub circRNA regulatory network based on hsa-miR-139-5p related subnetwork. The diamond, rectangle and ellipse indicated circRNA, miRNA and mRNA, respectively. Purple and blue represented up- and down-regulated, respectively. (H) Luciferase activity in cells co-transfected with luciferase reporter containing the wild-type or mutated miR-139-5p binding site of hsa_circ_007456 and miR-139-5p mimics or control. ^∗^*P* < 0.05 vs. control.

To assess whether the 4-DEmRNA signature has the potentiality to reflect the status of macrophage infiltration, the macrophage fractions of HCC cases with high or low risk score were further analyzed. The results showed that macrophage fractions of patients with high level of CDCA8, KPNA2, PRC1, TOP2A or risk score generated from 4-DEmRNA signature were significantly low both in TCGA (Figs. 7A–7E) and ICGC (Figs. S5A–S5E). In contrast, the macrophage infiltrations of HCC cases with low expression of miR-139-5p was significantly high (Fig. 7F). Based on the important role of 4-DEmRNA signature and miR-139-5p in macrophage infiltration and survival outcome, a hub circRNA-miRNA-mRNA regulatory network was constructed finally. This hub network contained 2 circRNA-miRNA pairs and 4 miRNA-mRNA pairs, including 2 circRNAs (hsa_circ_0007456 and hsa_circ_0091570), 1 miRNA (hsa-miR-139-5p) and 4 mRNAs (CDCA8, KPNA2, PRC1 and TOP2A) (Fig. 7G). Among this network, KPNA2 and TOP2A had been experimentally validated as the targeted genes of hsa-miR-139-5p (Hua et al., 2015; Pei, Yin & Liu, 2018; Zan et al., 2019). However, there was no report about these two circRNAs acting on hsa-miR-139-5p. We further used miRanda v3.3a, a microRNA target scanning algorithm, to predict the interaction between circRNA and miRNA. The results showed that the score of the interaction between hsa_circ_0007456 and hsa-miR-139-5p was 140, and the energy was -19.98 kCal/Mol, which is beneficial for hsa_circ_0007456 to function as hsa-miR-139-5p sponge. But there was no prediction result about the interaction between hsa_circ_0091570 and hsa-miR-139-5p. Thus, we choose hsa_circ_0007456 for investigation. The luciferase assay showed that the luciferase activity was inhibited when hsa-miR-139-5p mimics co-transfected with reporter plasmids containing the wide type sequence of hsa-miR-139-5p binding site in hsa_circ_0007456. While the luciferase activity had no significant change when co-transfection of hsa-miR-139-5p mimics and reporter plasmids containing the mutant sequence of binding site (Fig. 7H), indicating that hsa_circ_0007456 may act on hsa-miR-139-5p through miRNA sponge.

## Discussion

In this study, we used the EPIC tool to evaluate the different cell types in HCC cases from TCGA and ICGC projects. We found that patients with high fractions of total macrophages indicate better OS. HCC associated macrophages are derived from Kupffer cells and peripheral blood mononuclear cells, which play important roles in the occurrence and development of HCC (Weston, Zimmermann & Adams, 2019). Macrophages can be divided into different subtypes that play anti-cancer or cancer-promoting effects, which are related to the different prognosis of HCC patients (Dou et al., 2019; Rohr-Udilova et al., 2018; Tian et al., 2019) . For example, Kupffer cells can be divided into two types, namely CD68+MARCO- (macrophage receptor with collagen structure) and CD68+MARCO+cells (MacParland et al. 2018). The up-regulation of Marco expression inhibits the migration and invasion of HCC cells, thereby alleviating tumor progress (Sun et al., 2017). Furthermore, the massive infiltration of CD68+CD163+ macrophages and total CD68+ macrophages are related to the poor prognosis of HCC patients, while CD68+CD169+ macrophages and CD68+CD38+ macrophages are the opposite (Lam et al., 2019; Li et al., 2017). Unfortunately, the EPIC tool used in this study failed to subdivide the types of macrophages, and we could not further evaluate the role of macrophage subtypes and their circRNA regulatory networks in HCC. Future research may focus on the regulatory role of circRNA regulatory networks in macrophage infiltration and subtype switching.

By using WGCNA, 73 hub DEmRNAs were identified to be related to macrophage fraction and finally 27 DEmRNAs were included for circRNA network construction. All of the DEmRNAs in the network were correlated with the poor survival of HCC, indicating that these genes may play oncogenic roles. After co-expression and survival analysis, only the miR-139-5p was negatively co-expressed with some macrophage related DEmRNAs and showed the protective effects on the survival outcome for HCC. Similarly, expression of inflammatory and macrophage-related genes was opposite to decreased miR-139-5p in nonalcoholic fatty liver diseases samples (Latorre et al., 2017). Thus, CDCA8, KPNA2, PRC1 and TOP2A regulated by miR-139-5p were extracted as the hub subnetwork in this study. Although the role of these hub genes in the regulation of HCC related macrophages remains unknown, some of these genes have been reported to be correlated with the function of macrophage in other diseases. For instance, hydroxycholesterol inhibit miR-139-5p expression in macrophages to affect the function of osteoclast (Zhang et al., 2017). As for KPNA2, it mediates the nuclear import of SAMHD1 and contributes to the antiviral activity of human monocyte-derived macrophages (Schaller et al., 2014). Moreover, a previous study reported that PRC1 enhances the recruitment of regulatory T cells and M2-like tumor-associated macrophages, contributing to the metastasis of double-negative prostate cancer (Su et al., 2019). Tumor-associated macrophage is an essential factor affecting the efficiency of chemotherapy. Among the breast cancer patients with TOP2A overexpression, the absence of clinical response to anthracycline-containing neoadjuvant chemotherapy is associated with the presence of M2+ macrophage phenotype (Litviakov et al., 2018). In addition, TOP2A expression level was significantly negatively correlated with the numbers of macrophages in gastric cancer tissues (Zhang et al., 2020). These findings indicate that this subnetwork may be potentially involved in regulating the function of macrophage, which provides a promising direction for future mechanism study of HCC.

Previous studies have demonstrated that some miRNA-mRNA pairs of the hub subnetwork participate in the regulation of tumor progression. For instance, miR-139-5p is down-regulated in HCC tissues and overexpression of miR-139-5p inhibits HCC cell growth through suppressing KPNA2 (Zan et al., 2019). It was reported that miR-139-5p inhibits cell proliferation and migration by targeting TOP2A in pancreatic cancer and breast cancer cells (Hua et al., 2015; Pei, Yin & Liu, 2018). However, there are no reports about miR-139-5p acting on CDCA8 or PRC1 right now. As mentioned above, miR-139-5p and PRC1 have been demonstrated to be involved in the regulation of macrophage activity, which inspires us to suppose that upregulation of miR-139-5p may reduce the infiltration of M2 type macrophages by targeting PRC1 and attenuate the progression of HCC. Additionally, the result of our study was consistent with other findings that high level of miR-139-5p is correlated with a better prognosis of HCC (Hua et al., 2018; Wang et al., 2019a). Moreover, high level of miR-139-5p targeting DEmRNAs (CDCA8, KPNA2, PRC1, TOP2A) or risk score generated by 4-DEmRNA signature correlated with advanced clinical characteristics, poor survival and low macrophage infiltration level in HCC, while miR-139-5p showed the contrary result. Combining the above-mentioned low macrophage fractions suggesting a poor prognosis, it may explain the poor prognosis of HCC patients with low level of miR-139-5p or high level of targeted DEmRNAs to a certain extent.

Based on the important role of miR-139-5p regulatory subnetwork in the progression of HCC, we explored the candidate circRNAs acting on miR-139-5p. Finally, two circRNAs, namely hsa_circ_0007456 and hsa_circ_0091570, were screened out to construct the hub circRNA regulatory network. In this study, the luciferase assay result indicated that hsa_circ_0007456 has binding sites for miR-139-5p. A previous study has found that hsa_circ_0091570 functions as miR-1307 sponge to affect HCC cell proliferation, migration and tumor growth (Wang et al., 2019b). However, we did not further investigate the specific mechanism of these two circRNAs in modulating macrophage function related to HCC, which is a limitation of this study. Future study may focus on the mechanism in which circRNA modulates the macrophage activity to affect tumor microenvironment of HCC.

## Conclusions

We found that high macrophage infiltration level indicated good survival for HCC. A circRNA regulatory network was constructed based on macrophage-related DEmRNAs. Among this network, the expression of hsa-miR-139-5p was negatively correlated with CDCA8, KPNA2, PRC1 or TOP2A. Hsa-miR-139-5p low or targeted DEmRNA high expression was found to be correlated with low macrophage infiltration, high grade, advanced stage and poor prognosis of HCC. Additionally, the risk score generated by 4-DEmRNA signature could reflect the macrophage infiltration level and serve as an independent prognostic factor for HCC. Finally, the hub circRNA regulatory network was constructed based on hsa-miR-139-5p and four targeted DEmRNAs. Among this hub network, hsa_circ_0007456 was confirmed to act on hsa-miR-139-5p. Our findings indicate that certain circRNA regulatory network is potentially related to macrophage infiltration in HCC and provide a novel clue for the investigation of pathogenesis and therapeutic targets for HCC.

##  Supplemental Information

10.7717/peerj.10198/supp-1 Supplemental Information 1 Flow chart of the study design

10.7717/peerj.10198/supp-2 Supplemental Information 2 The Kaplan–Meier survival analysis of HCC cases with different miRNA expression level in TCGA

10.7717/peerj.10198/supp-3 Supplemental Information 3 Construction of 4-DEmRNA signature for predicting OS of HCC cases in ICGC (A) Comparison of CDCA8, KPNA2, PRC1 or TOP2A expression level between different stages. (B) Kaplan–Meier plot of OS of the signature for HCC. (C) The ROC curve of the signature for predicting 3-year survival rate of HCC. (D) Comparison of risk score between different stages. (E) The univariate and multivariate Cox regression analysis of risk score and other clinicopathological characteristics for HCC

10.7717/peerj.10198/supp-4 Supplemental Information 4 The Kaplan–Meier survival analysis and ROC curve predicting 3-year survival rate of HCC cases with different mRNA expression level in TCGA(A) and ICGC(B)

10.7717/peerj.10198/supp-5 Supplemental Information 5 The macrophage fractions in HCC cases with different expression levels of CDCA8 (A), KPNA2 (B), PRC1 (C), TOP2A (D) or risk score (E) from ICGC

10.7717/peerj.10198/supp-6 Supplemental Information 6 Raw data

## Additional Information and Declarations

### Data Availability

The following information was supplied regarding data availability: The raw measurements are available in the Supplemental Files.
