# Supplementary figures and images for: Construction and analysis of macrophage infiltration related circRNA-miRNA-mRNA regulatory networks in hepatocellular carcinoma
Source: PeerJ. 2020 Oct 20;8:e10198. doi: 10.7717/peerj.10198 (PMC7583625; doi:10.7717/peerj.10198)

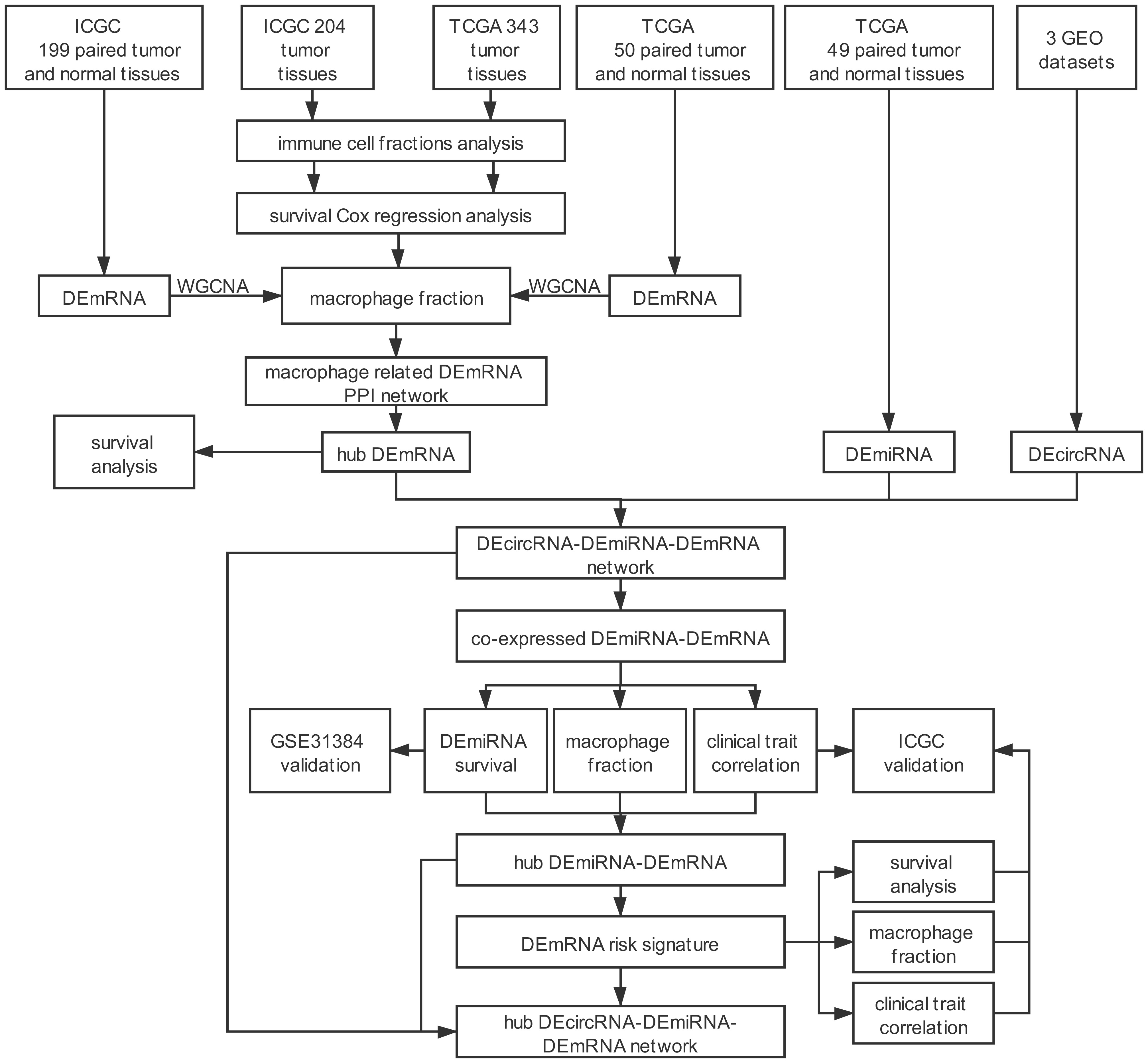

Supplement: Supplemental Information 1 [file peerj-08-10198-s001.png]

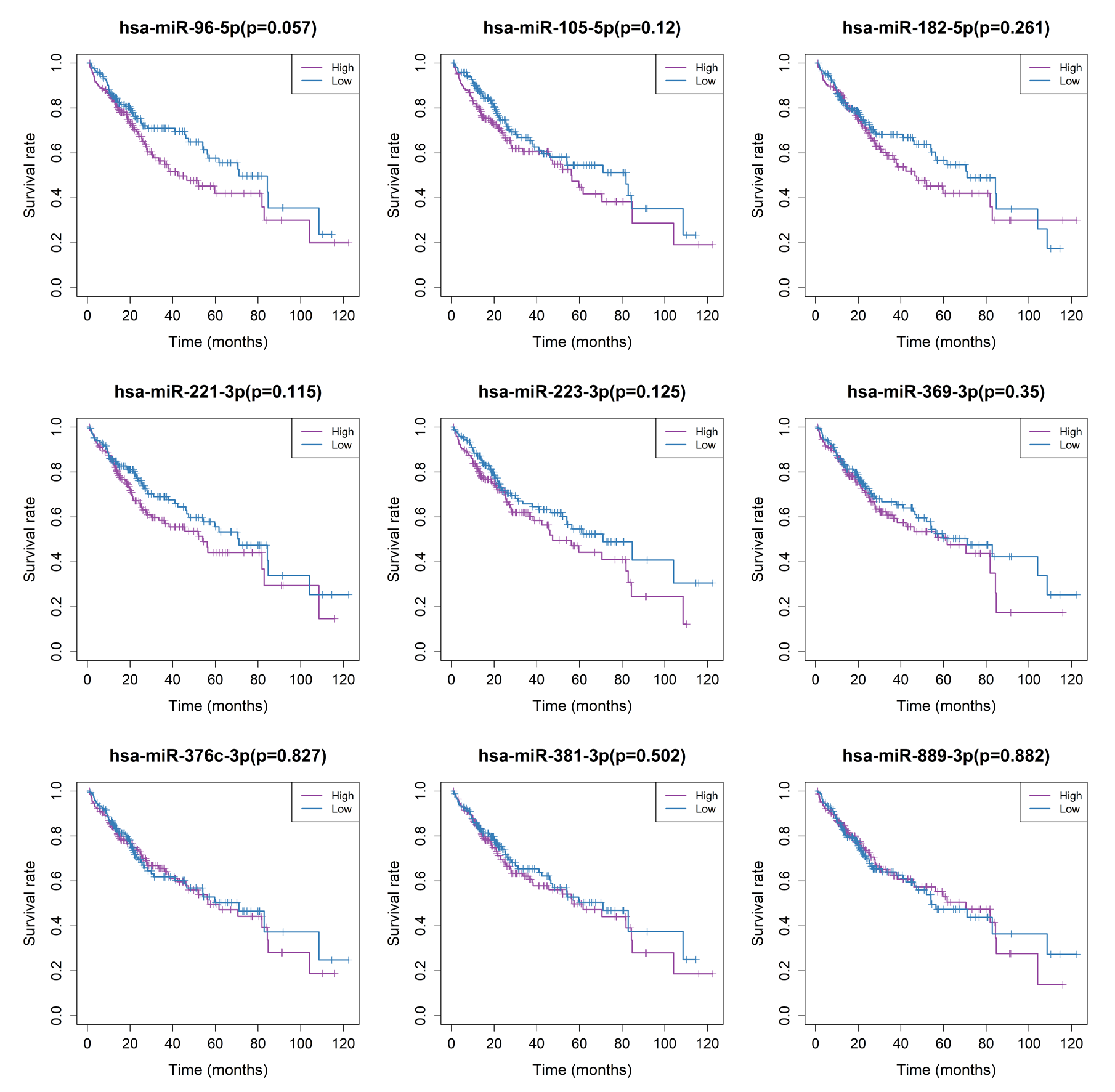

Supplement: Supplemental Information 2 [file peerj-08-10198-s002.png]

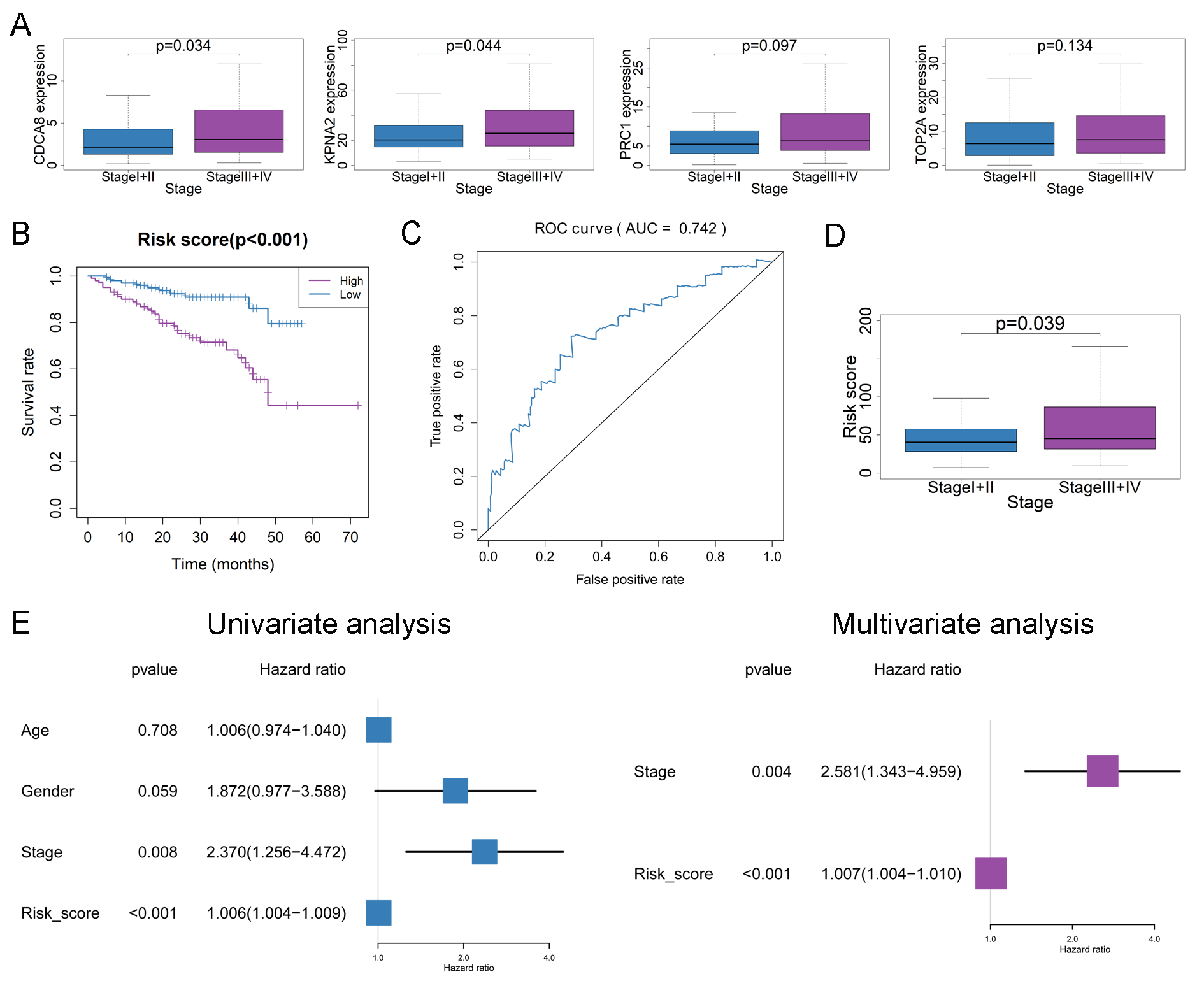

Supplement: Supplemental Information 3 — (A) Comparison of CDCA8, KPNA2, PRC1 or TOP2A expression level between different stages. (B) Kaplan–Meier plot of OS of the signature for HCC. (C) The ROC curve of the signature for predicting 3-year survival rate of HCC. (D) Comparison of risk score between different stages. (E) The univariate and multivariate Cox regression analysis of risk score and other clinicopathological characteristics for HCC [file peerj-08-10198-s003.png]

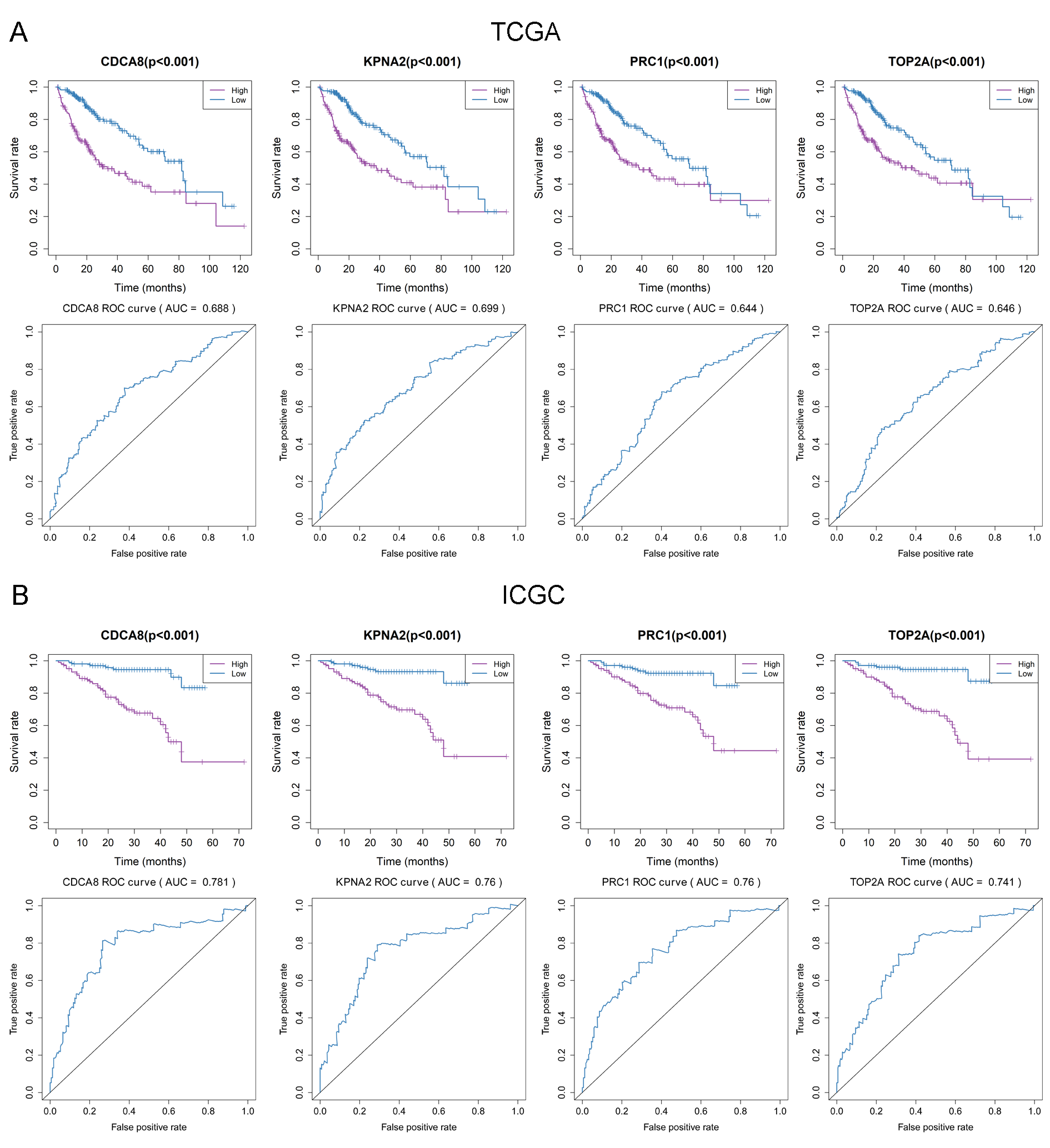

Supplement: Supplemental Information 4 [file peerj-08-10198-s004.png]

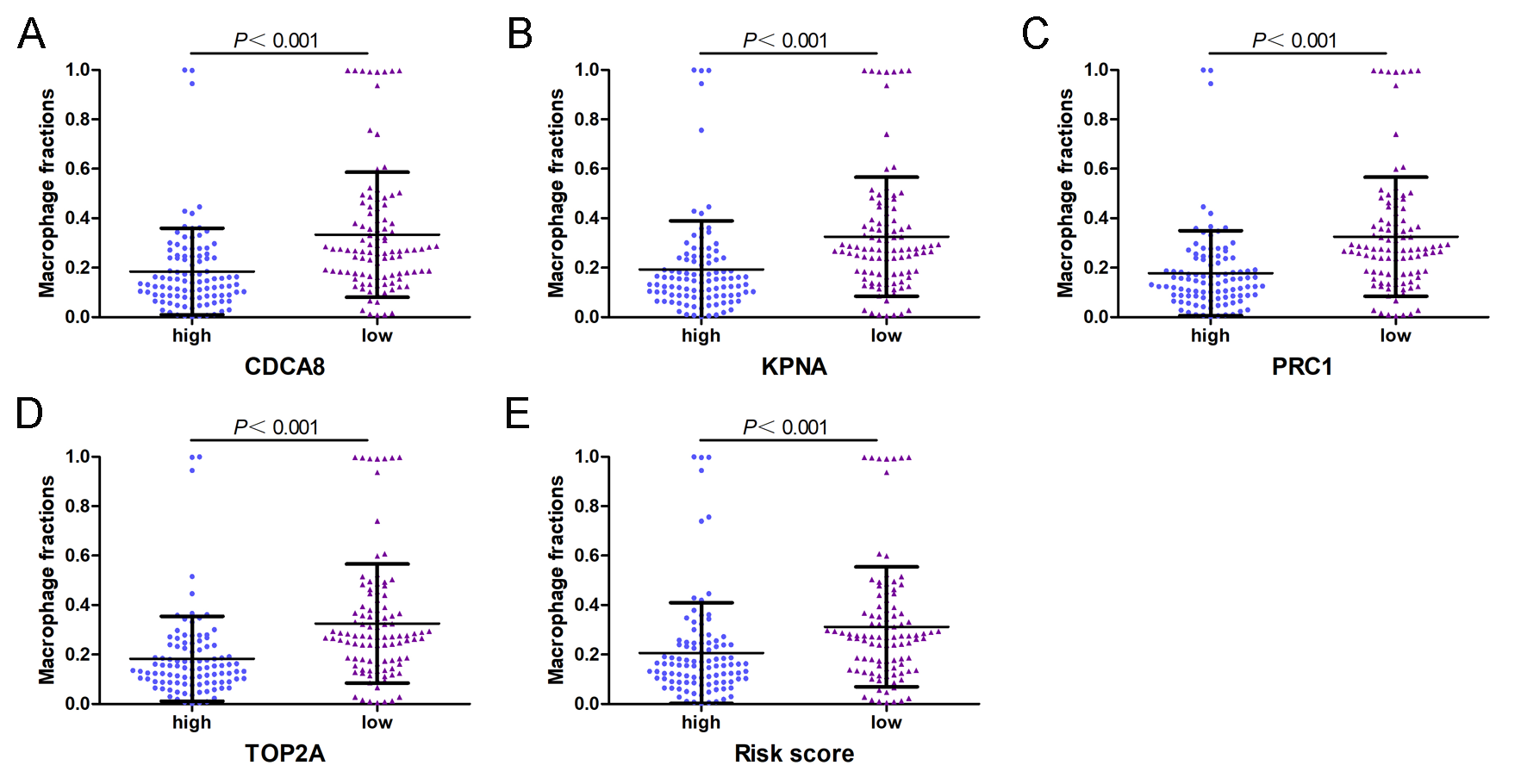

Supplement: Supplemental Information 5 [file peerj-08-10198-s005.png]
